# Transactions of American Medical Association

**Published:** 1865-09

**Authors:** 


### Transactions of American Medical Association.

Members who desire copies of the Transactions for 1865, must forward their subscriptions, ($3,) immediately, as the edition will be limited to the number required at the time of going to press.

WM. B. ATKINLON,
Perm. Sec'y, Philadelphia.

As there are various methods by which the volume may be sent, inform me which you prefer. If by MAIL, please forward THIRTY-TWO CENTS IN POST-OFFICE STAMPS, that your postage may be prepaid.

Very respectfully,
CASPAR WISTER,
Treas. Amer. Med. Association,
No. 1303 Arch street.

